# Therapeutic role of mesenchymal stem cells and platelet-rich plasma on skin burn healing and rejuvenation: A focus on scar regulation, oxido-inflammatory stress and apoptotic mechanisms

**DOI:** 10.1016/j.heliyon.2023.e19452

**Published:** 2023-08-24

**Authors:** Bakinam M.H. Tammam, Ola A. Habotta, Manal El-khadragy, Ahmed E. Abdel Moneim, Mohga S. Abdalla

**Affiliations:** a Chemistry Department, Molecular biotechnology Division, Faculty of Science, Helwan University, Cairo, Egypt; b Department of Forensic Medicine and Toxicology, Faculty of Veterinary Medicine, Mansoura University, Mansoura, Egypt; c Biology Department, Faculty of Science, Princess Nourah Bint Abdulrahman University, P.O. Box 84428, Riyadh 11671, Saudi Arabia; d Zoology and Entomology Department, Faculty of Science, Helwan University, Cairo, Egypt

**Keywords:** Angiogenesis, Burn healing, Mesenchymal stem cells, Oxidative stress and inflammation, Platelet-rich plasma, Re-epithelialization

## Abstract

Cell-based therapies have great promise in accelerating and improving burn wound healing. It is a growing need to scale their competence to meet the clinical demands. In this study, the bone marrow mesenchymal stem cells (BMSCs) and platelet-rich plasma (PRP) were tested on the repair of induced burn wounds in a murine model. After the induction of thermal injury, rats were injected with BMSCs and/or PRP in the burn area. After 4 weeks of post-burn, our findings revealed that local treatment of burnt skin with BMSCs and/or PRP offered substantial outcomes when compared with the untreated group. Injected burn with BMSCs and/or PRP enhanced the wound contraction rate and decreased the burn area and period of epithelization. Significant increases in VEGF together with declines in MMP-9 and TGF-β1 were observed in burnt areas after being treated with BMSCs and/or PRP therapy that indicated improved angiogenesis, and re-epithelization. Furthermore, both MSCs and PRP modulated the burn's oxidative and inflammatory microenvironment as indicated by increases in SOD, CAT, and GSH besides declines in MDA, IL-6, TNF-α, NF-κB, NO, and iNOS. Notable increases in Bcl-2 levels and decreases in Cas-3 and Bax levels were recorded in burnt skin that received both agents concomitantly. Interestingly, the histopathological examination validates the healing power of BMSCs and/or PRP. Collectively, BMSCs and PRP have pioneered therapeutics candidates for clinical application in burn healing possibly via antioxidant, anti-inflammatory, and anti-apoptotic mechanisms along with regulating angiogenesis and scar formation.

## 1 Introduction

One of the most prevalent injuries in the world is burnt. When the skin is subjected to heat, radiation, electricity, or chemicals, burns develop [1]. Burned skin is extremely at risk for bacteria and other pathogens, due to the loss of layers of skin protection. The skin has three main layers namely the epidermis, dermis, and hypodermis and they play an important role where functioning ensures homeostasis and protects against aggressive and causative agents in the environment [2]. Burns are classified by degrees depending on how deeply and harshly they penetrate the skin's surface. The first degree is red and blistered skin in the epidermal layer of the skin, the second degree is blistered and thickened in the epidermal and dermal layers of the skin, and the third degree has widespread thickening with a white leathery appearance where the damage has affected the epidermis, dermis, hypodermis and reached the muscles and nerves [3]. Homeostasis (coagulation), inflammation (mononuclear cell infiltration), proliferation (epithelialization, fibroplasia, angiogenesis, and granulation tissue development), and maturation (scar tissue development or collagen deposition) are the four ordinary phases of the systematic process of skin healing [4].

After burn injuries, a number of factors, including the causes, the extent and location of the burn, the patient's overall health, and the types of graft or material used to cover the burn, affect how the skin heals [5]. In comparison to other wound types, burns have systemic consequences; practically all bodily systems are impacted, whether through changes in the heart, kidney, liver, lung, bone marrow, or lymphoid organ [6]. The inflammatory mediator that is responsible for systematic effects is released such as tumor necrosis factor-alpha (TNF-α) and interleukins 6, 8, and 1β. The concentration of inflammatory mediators in serum correlated with the burn surface area. If the concentration increases the risk of infections increases and the syndrome of numerous organ malfunction and demise [7].

It has been shown through recent studies that direct injection of bone marrow mesenchymal stem cells (BMSCs) has a promising potential in the complex process of wound healing through their promising self-renewal, differentiation potential, and low immunogenicity profile. There are two known mechanisms of BMSCs that propagate the skin regeneration process: The first mechanism is conditioned on the ability of the BMSCs to differentiate into numerous skin cells types such as keratinocytes and endothelial cells [8,9]. The second mechanism is due to the ability of BMSCs-related paracrine activity to initiate the release of growth factors, cytokines, and extracellular vesicles [10].

It has also been depicted in recent studies that platelet-rich plasma (PRP) has a beneficial role in accelerating and enhancing burn wound healing. PRP is highly concentrated plasma with platelet drawn from blood. When platelets get activated, they discharge many growth factors such: as platelet-derived growth factors (PDGF), epidermal growth factor (EGF), transforming growth factor β (TGF-β), vascular endothelial growth factor (VEGF), fibroblast growth factor (FGF), and insulin-like growth factor (IGF). In multiple ways, these growth factors affect burn wound healing by promoting chemotaxis, cell adhesion, mitogenesis, proliferation, and angiogenesis [11].

Recent studies showed that combining PRP and BMSCs could increase the effectiveness of wound healing, and enhance the regeneration and differentiation of skin cells [12]. This combination therapy has demonstrated superiority over monotherapy using BMSCs or PRP only in terms of shortening the wound healing duration [13]. Through the guidance of PRP to the BMSCs by secreting growth factors that differentiate stem cells (SC) to the target cells which ameliorate the process of healing [13].

## 2 Materials and methods

### 2.1 Animals and experimental ethics statement

Thirty-five male Wistar albino rats were obtained from VACSERA, Giza, Egypt. They weighed between 200 and 220 g and had an average age of 10 weeks. They were kept in wire polypropylene cages with a 12-h light/dark cycle before the experiment. For two weeks, they were kept in a controlled environment with temperatures of 25 2 °C and relative humidity levels of 55 15%. Each rat was fed a standard meal and had unrestricted access to water. The European Community Directive (86/609/EEC) was followed in conducting the current experimental method. The Institutional Animal Ethics Committee accepted the animal care procedures at the Zoology and Entomology Department, Faculty of Science, Helwan University, and they complied with the National Institutes of Health (NIH) Guidelines for the Care and Use of Laboratory Animals, 8th edition. (Approval Number: HU2021/Z/AEB1221-01).

### 2.2 Isolation and culture of bone marrow mesenchymal stem cells (BMSCs)

BMSCs were isolated using the methods previously mentioned [14]. Briefly, three 4-week-old rats were killed by cervical dislocation under anaesthesia with a dose of pentobarbital (300 mg/kg) injected intraperitoneally (i.p.). Rat tibias and thighs were separated, and all of the muscles that were linked were cut off. After inserting a 3 mL syringe needle into the bone hole, bone marrow was aspirated, and then 1 mL culture media was repeatedly pumped through the bone until all of the bone marrow cells had been removed. The remaining material was filtered out before the bone marrow cells were dispersed into a single-cell suspension. The suspended cells were grown in Dulbecco's Modified Eagle Medium (Sigma, St. Louis, MO, USA) containing low glucose, 10% foetal bovine serum (FBS), 100 IU/mL streptomycin, and a combination of 100 IU/mL penicillin and streptomycin. The non-adherent cells were removed after a 24-h culture at 37 °C to a 5% CO_2_ environment, and the medium was renewed every 72 h.

### 2.3 Preparation of PRP

To separate the buffy coat that containing platlates, the whole blood 10 mL was centrifuged at 220×*g* for 20 min (at room temperature) with anticoagulant dextrose solution A (ACD-A) at a ratio of 9:1. The buffy coat was taken out by pipette and transferred into a sterile citrate-free vacuette. For 20 min, the solution was centrifuged once more at 480×*g* to isolate the PRP with the highest concentration of platelets. The PRP was activated by adding a thrombin activator (500 U bovine thrombin in 1 mL 10% calcium chloride) [15,16]. The collected PRP was counted by using disposable counting hemocytometers.

### 2.4 The burning step

All rats received the burn. All rats underwent a 6-h starvation period with free access to water prior to the operation. Ketamine hydrochloride (50 mg/kg) was intraperitoneally injected to provide them with anaesthesia. In addition, the animals received a single dosage of ketoprofen (2.5 mg/kg, subcutaneously injected) as a morally acceptable method of reducing postoperative discomfort. The skin surface of each rat was first sanitized with 70% alcohol after the hair on its back was removed.

Secondly, a 100 g cylindrical stainless-steel rod (1 cm diameter) was heated to 100 °C in boiling water. The rats get exposed to the hot stainless-steel rod for 3 s. Afterward, rats were placed individually (each group per cage) and appropriately monitored in sterile cages to avoid infection [17].

### 2.5 Animal groups and sampling

To evaluate the healing potential of BMSCs and PRP, rats were divided into five groups (n = 7) as shown below:

Group I: represented the untreated control group. In this group, the burn was covered only with a sterile dressing.

Group II: represented the traditional treatment group. Rats were daily treated with 10% w/w povidone-iodine solution (PIS; Pharaonia Pharmaceuticals, Borg Alarab, Alexandria, Egypt; reference drug) and applied topically once a day.

Group III: rats treated with BMSCs (1 × 10^5^ cells) injected directly in the burn area once 24 h after the burn procedures. The number of BMSCs used in the present work was based on the pervious study of Al-Otaibi et al. [14].

Group IV: rats treated with PRP (4.8 × 10^6^ platelets) injected directly into the burn area once time 24 h after the burn procedures. The number of platelets used in the current study was based on the pervious work of Chen et al. [18].

Group V: rats treated with a combination of BMSCs and PRP injected directly in the burn area once 24 h after the burn procedures at the same above mentioned doses.

After 28 days of healing of the burn region, all animals utilized for research were euthanized by cervical dislocation under pentobarbital anaesthesia (300 mg/kg i.p.). For the appropriate analysis, samples of skin tissue and blood were taken. The skin tissue from the burnt region was separated into three sections. The first section was used to prepare tissue homogenate (10% w/v), which was then centrifuged at 3000 g for 10 min at 4 °C after being mixed with an ice-cold medium containing 50 mM Tris-HCl (pH 7.4). The biochemical analysis was then performed using the acquired supernatants. The second portion was kept at −80 °C for use in gene expression analysis, while the third portion was fixed in formalin for use in histopathological H&E and Sirius Red staining as well as NF-κB and TGF-β immunohistochemistry.

### 2.6 Assessment of tissue-remodeling biomarkers

Transforming growth factor beta (TGF-β; Catalogue number: MB200) and matrix metalloproteinase-9 (MMP-9; Catalogue number: RMP900) were measured in skin tissue by ELISA kits obtained from R&D Systems (Minneapolis, MN, USA) in accordance with the manufacturer's instructions. MMP-9 is a key mediator of acute inflammation, while TGF-β is a regulator of the burn healing process.

### 2.7 Measurement of angiogenesis marker in burnt skin

The level of vascular endothelial growth factor (VEGF; Catalogue number: RRV00) was analyzed using an ELISA kit according to the R&D Systems manufacturer's protocol.

### 2.8 Oxidative stress markers

Malondialdehyde (MDA), a secondary LPO product, was measured using spectrophotometry in burnt skin homogenates to measure lipid peroxidation (LPO) by utilizing the method of Ohkawa [19]. By reducing 5,5′-dithiobis (2-nitrobenzoic acid) to yellow 5-thionitrobenzoic acid, glutathione (GSH) was measured spectrophotometrically at 405 nm [20].

### 2.9 Enzymatic antioxidant status

The capacity of superoxide dismutase (SOD) to prevent the nitroblue tetrazolium dye from being reduced to diformazan after being begun by phenazine methosulphate was used to test the enzyme's activity (PMS) [21]. Based on the depletion of hydrogen peroxide (H_2_O_2_) into water and oxygen at 240 nm, catalase (CAT) activity was calculated [22].

### 2.10 Inflammatory biomarkers

Using ELISA kits (R&D System, Minneapolis, MN, USA), the levels of TNF-α and IL-6 in the burnt skin homogenates were measured in accordance with the manufacturer's instructions. Using an ELISA kit from Active Motif North America in Carlsbad, California, and adhering to the manufacturer's instructions, the NF-κB DNA binding activity was assessed in the nuclear fraction. The NF-κB p65 (ng/mg protein) activity in the samples was used to reflect the activities. Nitric oxide (NO) was also measured by colorimetry using Griess reagent for nitrite/nitrate [23].

### 2.11 Measurement of apoptosis-related biomarkers

ELISA kits (Biospes, China) were used to test the quantities of Bax and Bcl-2 proteins in triplicate, in accordance with the manufacturer's instructions. Briefly, the ELISA kit's instructions were followed to lyse treated and untreated cells. Specific Bax and Bcl-2 proteins from the cell lysate bound to the main antibody and were found by secondary antibodies that were HRP-conjugated. Subsequently, using a microplate reader to measure the absorbance at 450 nm, the levels of the proteins Bax and Bcl-2 were measured. The caspase-3 family (CPP-32 like) appears to play a dominant role in the downstream signaling of apoptotic death, to establish the signal transduction pathway inducing apoptosis after burn injury, we examined caspase-3 activity and its inhibition on apoptosis. The Cas-3 was measured using an ELISA kit according to the manufacturer's protocol.

### 2.12 Gene expression analysis

The TRIzol reagent (Life Technologies, Gaithersburg, MD, USA) was used to isolate total RNA from the skin in the burn region in accordance with the manufacturer's instructions. Following that, cDNA was created right away using the MultiScribe RT enzyme kit (Applied Biosystems, Foster City, CA, USA). Real-time PCR analysis was performed in triplicate on the acquired cDNA. Power SYBR Green Master Mix (Bio-Rad, CA, USA) was used in real-time PCR experiments using a 7500 Real-Time PCR System (Applied Biosystems, Foster City, CA, USA).

The PCR analysis's thermal cycle consisted of 40 cycles at 95 °C for 10 s each, followed by 60 °C for 30 s and 72 °C for 10 s. The relative fold change in the iNOS, Cas-3, Bax and TGF gene's mRNA expression was calculated and assessed against the control. The reference housekeeping gene glyceraldehyde-3-phosphate dehydrogenase (GAPDH) was used. Primer sequences and accession numbers are provided in Table 1.

**Table 1:** Primer sequences of genes analyzed in real time PCR.

Name	Accession number	Forward primer (5'---3′)	Reverse primer (5'---3′)
GAPDH	NM_017008.4	CTCTCTGCTCCTCCCTGTTC	TACGGCCAAATCCGTTCACA
TGFβ	NM_013174.2	TCACACAGCGCAGTGAGTTC	GAAAGGGGGAGGAAAACCAGG
iNOS	NM_012611.3	AGTCAACTACAAGCCCCACG	GCAGCTTGTCCAGGGATTCT
Bax	NM_017059.2	CACGTCTGCGGGGAGTCAC	TAGAAAAGGGCAACCACCCG
Cas-3	NM_012922.2	GAGCTTGGAACGCGAAGAAA	TTGCGAGCTGACATTCCAGT

### 2.13 Histopathological examination

Burned tissues were dehydrated in escalating grades of ethanol, cleaned with xylene, and embedded in molten paraplast before being fixed in 10% neutral buffered formalin for 24 h. Hematoxylin and eosin were used to stain the finished blocks, which were divided into pieces that were 4–5 m thick. A Nikon microscope (Eclipse E200-LED, Tokyo, Japan) was used to examine tissue slices at the microscopic level. For the purpose of observing the deposition of collagen fibres, additional sections were dyed with Sirius Red.

### 2.14 Immunohistochemical examination

Tissue sections were examined for the immune reaction of NF-κB and TGF-β in all treated groups. Tissue blocks were deparaffinized and rehydrated in consecutive graded ethyl alcohol solutions. Antigen was retrieved by EDTA solution, pH 8 and the inactivation of endogenous peroxidases was done by utilizing a hydrogen peroxide solution for 5 min. After that, the slide was incubated for 1 h with primary-monoclonal antibody for preventing non-specific binding. Following, the slide was washed up with PBS and incubated with anti-rat IgG secondary antibodies (1:1000 dilution) for 10 min. Finally, the brown stain was evident with DAB substrate and counterstained was with Mayer's hematoxylin for 10 min.

### 2.15 Statistical analysis

The mean and standard deviation (SD) were used to express all values. To ascertain the significance between groups, collected data were subjected to one-way analysis of variance (ANOVA) analysis, followed by the post hoc Duncan's multiple range test. With *P* values less than 0.05, differences were deemed statistically significant.

## 3 Results

### 3.1 PRP or/and BMSCs enhanced the healing, contracture, and re-epithelization of burnt tissues

**Fig. 1:**
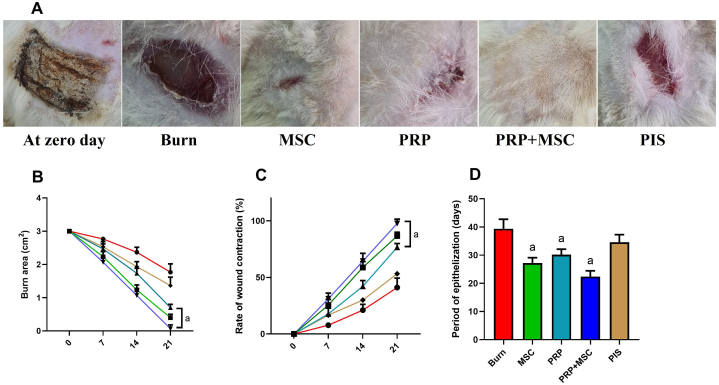
Effects of bone marrow derived-mesenchymal stem cells (BMSCs), platelet-rich plasma (PRP), BMSCs + PRP, or standard treatment (PIS) on **(A and B)** burn area, **(C)** rate of burn contraction (%), and **(D)** period of epithelization (days) in a full-thickness skin burns in rats. The results are presented as mean ± SD values (n = 7). The letters (^a^ and ^b^) indicate statistical differences at *P* < 0.05 in comparison with the control and the model groups, correspondingly.

Following the skin thermal injury, the healing status and the ability of hair growth were determined as shown in Fig. 1-A. The results revealed a gradual decline in the original burn area over time in all studied groups. On the examined days post-burn, significant reductions of the burn of 3 groups (BMSCs, PRP, and PRP + BMSCs) were observed compared to the untreated control group or treated with traditional treatment (PIS group) (Fig. 1-B). Furthermore, our findings indicated that the therapeutic intervention either with PRP, BMSCs, or both significantly enhanced the burn contraction rate on days 7, 14, 21, and 28 post-burning related to the untreated burnt rats (Fig. 1-C). Besides, the days of the epithelialization phase were recorded in treated and untreated burned rats. The epithelialization time in burned rats treated with PRP, BMSCs, or PRP/BMSCs was significantly faster than (*P* < 0.05) those when compared to the untreated burned tissues. Notably, no significant difference was noticed in the epithelialization period in burns treated with the traditional treatment in relation to untreated burns (Fig. 1-D).

### 3.2 BMSCs along with PRP enhanced the remodeling-associated biomarkers in burnt skin

**Fig. 2:**
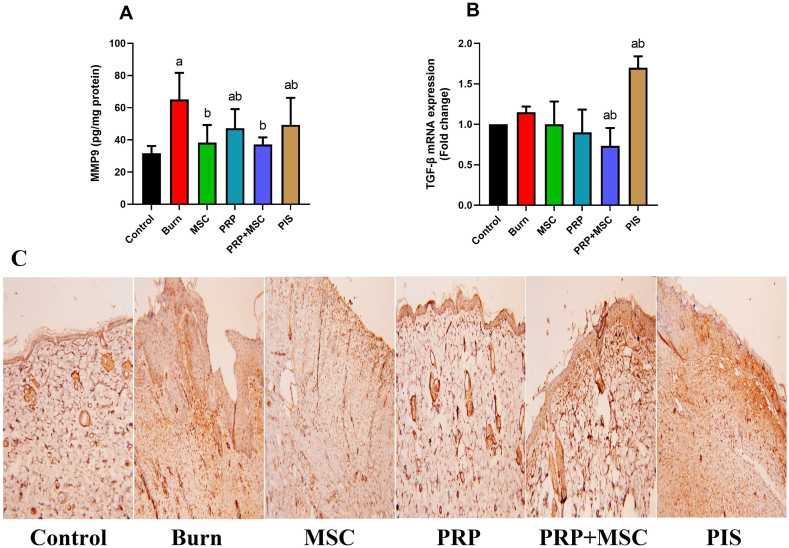
Effects of bone marrow derived-mesenchymal stem cells (BMSCs), platelet-rich plasma (PRP), BMSCs + PRP, or standard treatment (PIS) on **(A)** matrix metalloproteinase (MMP9) level and **(B)** transforming growth factor beta (TGF-β) mRNA expression in a full-thickness skin burns in rats. TGF-β was also examined immunohistochemically as shown in palate **C** (100x). Results of biochemical assays are expressed as mean ± SD values (n = 7), whereas, gene expression results are expressed as the mean ± SD of triplicate assays and adjusted to GAPDH. The letters (^a^ and ^b^) indicate statistical differences at *P* < 0.05 in comparison with the control and the model groups, correspondingly.

As illustrated in Fig. 2-A, the burn group had higher levels (*P* < 0.05) of MMP-9 than those of the control group. However, the treated burnt animals with different treatments presented significant reductions (*P* < 0.05) in this marker when compared with the untreated burn group. Remarkably, the decrease of the MMP-9 levels in groups that received BMSCs alone or combined with PRP was close to the values of the control one that indicating their potential contribution to the proliferation and remodeling of the burnt skin. Furthermore, the tissue levels, gene expression, and immune reaction of TGF-β, another key marker for the efficacy of the healing process, were measured in burnt tissue (Fig. 2-B). The results revealed that the burnt tissue represented significant increases (*P* < 0.05) in TGF-β in comparison to the control. On contrary, its values were markedly decreased (*P* < 0.05) were noticed in burnt tissue upon being treated with BMSCs, PRP, or both. Notably, the traditional therapy with PIS had no effect on tissue TGF-β levels. In the same context, the immunohistochemical analysis validated the positive effect of different treatments on this biomarker in burnt tissues especially with PRP and combined treatment (Fig. 2-C).

### 3.3 BMSCs and PRP synergistically promoted the angiogenesis process in burnt skin

**Fig. 3:**
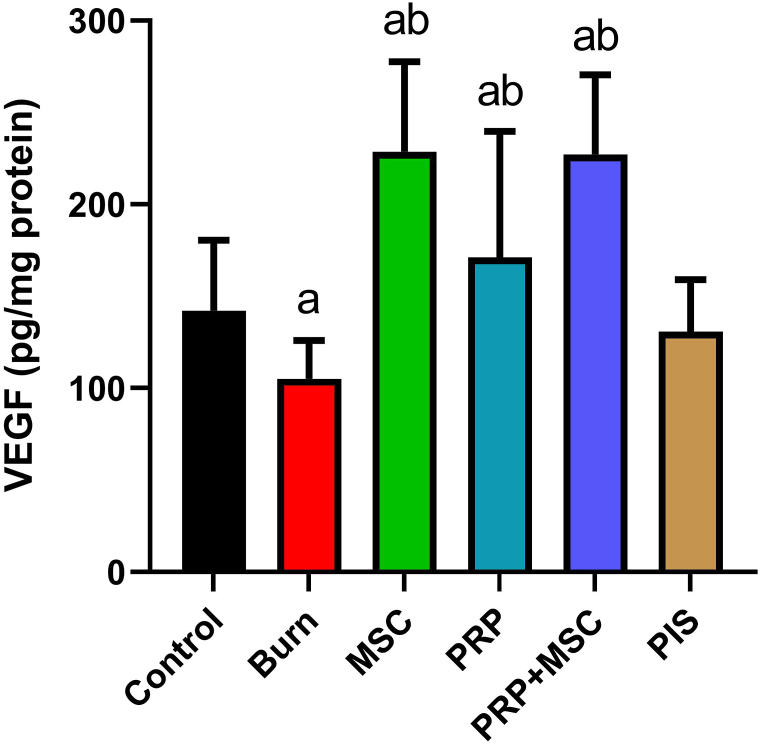
Effects of bone marrow derived-mesenchymal stem cells (BMSCs), platelet-rich plasma (PRP), BMSCs + PRP, or standard treatment (PIS) on vascular endothelial growth factor (VEGF) levels in a full-thickness skin burns in rats. The results are presented as mean ± SD of triplicate assays and adjusted to GAPDH. The letters (^a^ and ^b^) indicate statistical differences at *P* < 0.05 in comparison with the control and the model groups, correspondingly.

As a sensitive angiogenesis biomarker, the mRNA gene expression of VEGF was measured after 28 days post-burn in the treated and untreated burn tissues. When compared to the control, significant increases (*P* < 0.05) were observed in the burn tissue. In contrast, the burnt groups that received PRP, BMSCs, or PRP + BMSCs displayed marked increases (*P* < 0.05) in the gene expression of this marker the untreated burnt group (Fig. 3). In addition, the PIS treatment was not able to induce any difference when compared to the model group.

### 3.4 Combined BMSCs and PRP therapy relieved the tissue oxidative stress in burnt skin

**Fig. 4:**
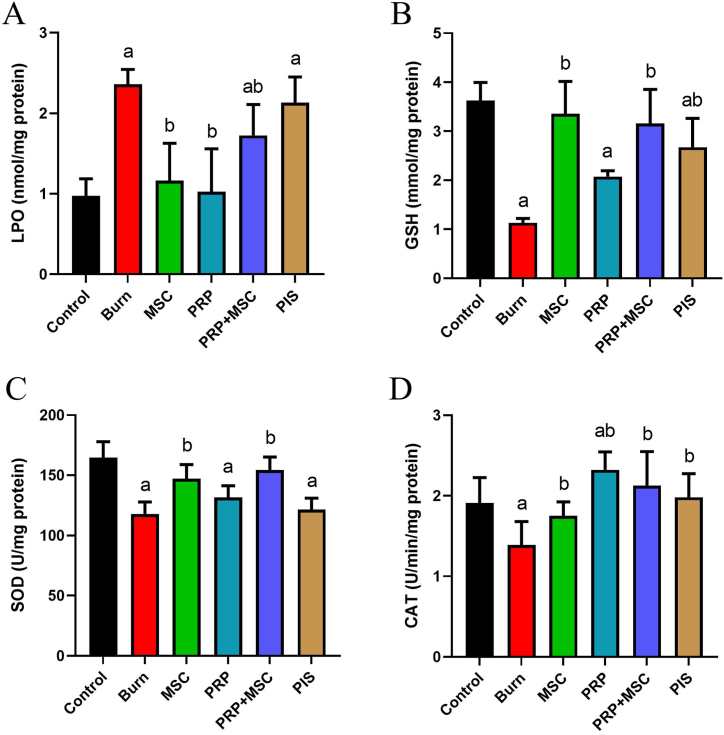
Effects of bone marrow derived-mesenchymal stem cells (BMSCs), platelet-rich plasma (PRP), BMSCs + PRP, or standard treatment (PIS) on the levels of enzymatic (SOD and CAT) as well as non-enzymatic oxidative stress markers (GSH, and MDA) in full-thickness skin burns in rats. The results are presented as mean ± SD values (n = 7). The letters (^a^ and ^b^) indicate statistical differences at *P* < 0.05 in comparison with the control and the model groups, correspondingly. A: LPO level, B: GSH level, C: SOD activity, and D: CAT activity.

The oxidant/antioxidant status in burnt in response to the thermal injury and different therapies is shown in Fig. 4. Remarkable decreases (*P* < 0.05) were detected in the activities of SOD and CAT, as well as the content of GSH in tissues exposed to the thermal injury in respect to the control. Besides, the lipid peroxidation was markedly elevated in the model group as indicated by higher MDA levels (*P* < 0.05) than those of the control group. Adversely, BMSCs, PRP, PRP/BMSCs, or PIS treatments significantly enhanced (*P* < 0.05) the CAT activities and lessened the MDA levels compared to the untreated burn group. In addition, BMSCs alone or combined with PRP were able to restore GSH levels and SOD activities (*P* < 0.05) in the burnt tissue.

### 3.5 Concomitant BMSCs and PRP therapy subsided the inflammation response in burnt tissue

**Fig. 5:**
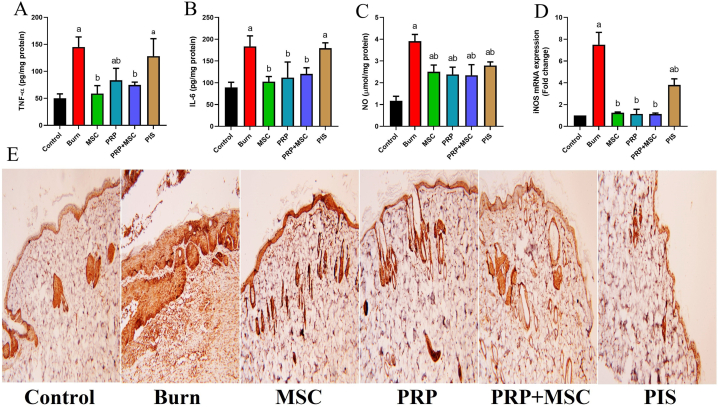
Effects of bone marrow derived-mesenchymal stem cells (BMSCs), platelet-rich plasma (PRP), BMSCs + PRP, or standard treatment (PIS) on the (upper panel) inflammatory biomarkers (TNF-α, IL-6, and NO) and the mRNA expression levels of iNOS in full-thickness skin burns in rats. (E): NF-κB was also detected immunohistochemically (lower panel) on skin sections (100x). The results are presented as mean ± SD values (n = 7). The letters (^a^ and ^b^) indicate statistical differences at *P* < 0.05 in comparison with the control and the model groups, correspondingly. A: TNF-α level, B: IL-6 level, C: NO level, D: iNOS expression and E: NF-κB immunohistochemical photos.

Since the significant contribution of inflammatory mediators in the progress and relief of burn wounds, their levels were measured in different groups (Fig. 5). The obtained results detected that the induction of burn evoked markedly higher levels (*P* < 0.05) of TNF-α, and IL-6 than those in the control group. In addition, we observed substantial increases (*P* < 0.05) in the levels and the immune reaction of NF-κB, a crucial inflammatory regulator, in burn-stressed tissue. On the other side, BMSCs, PRP, or their combination notably subsided these mediators and tissue inflammatory reaction (*P* < 0.05) in the model group, while non-significant change was observed with the PIS therapy. Also, the elevated cytokines levels induced the iNOS expression as demonstrated by upregulated (*P* < 0.05) gene expression and NO levels in tissues with burn injury. All treatments had the efficacy to suppress (*P* < 0.05) the iNOS mRNA expression with subsequent declines in the NO levels with respect to the model group.

### 3.6 BMSCs and PRP synergistically relieved the tissue apoptosis in burnt skin

**Fig. 6:**
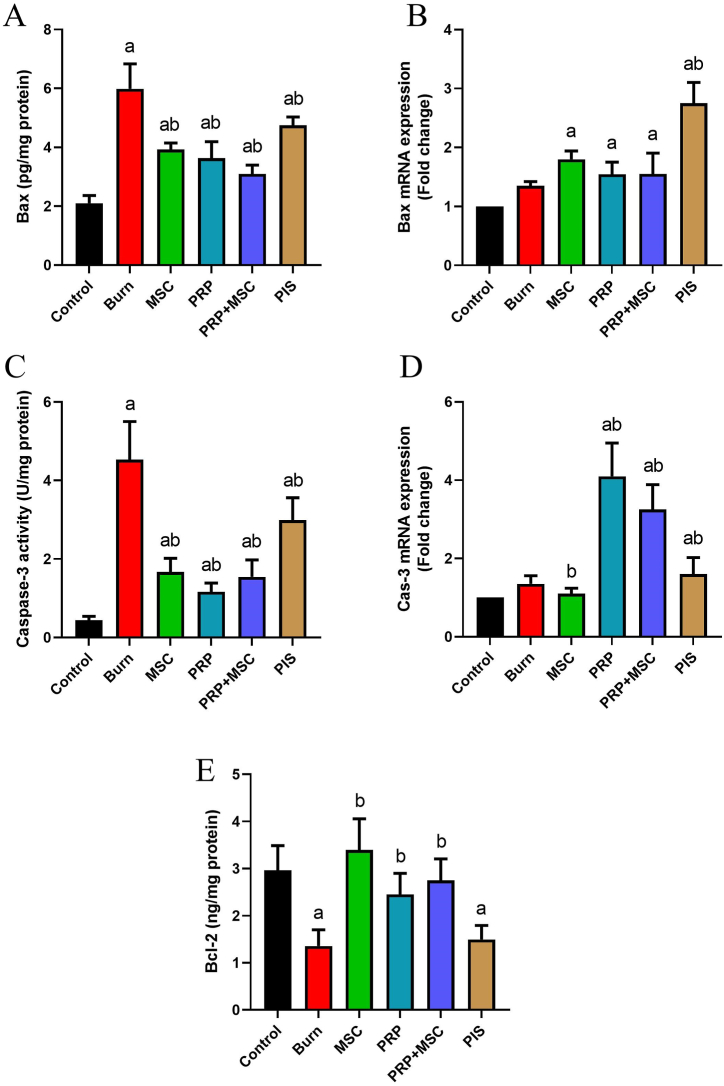
Effects of bone marrow derived-mesenchymal stem cells (BMSCs), platelet-rich plasma (PRP), BMSCs + PRP, or standard treatment (PIS) on the levels of apoptotic markers (Bax, Cas-3, and Bcl-2) levels in a full-thickness skin burns in rats. Results of biochemical assays are expressed as mean ± SD values (n = 7), whereas, gene expression results are expressed as the mean ± SD of triplicate assays and adjusted to GAPDH. The letters (^a^ and ^b^) indicate statistical differences at *P* < 0.05 in comparison with the control and the model groups, correspondingly. A and B: Bax level and expression, C and D: Caspase-3 activity and expression, and E: Bcl-2 level.

The apoptosis-associated markers in the burn injury of different groups are displayed in Fig. 6. Noteworthy increases (*P* < 0.05) were recorded in the levels of pro-apoptotic markers (Cas-3 and Bax) together with decreases (*P* < 0.05) in the anti-apoptotic marker (Bcl-2) levels in the group with burn injury. Nevertheless, the injured groups that received BMSCs, PRP, or BMSCs/PRP showed significant decreases (*P* < 0.05) in Cas-3 and Bax levels as well as increases in Bcl-2 levels. PIS treatment also lessened (*P* < 0.05) the pro-apoptotic markers without any observable effect on Bcl-2 (Fig. 6).

### 3.7 BMSCs and PRP restored the normal skin histology in burnt area

**Fig. 7:**
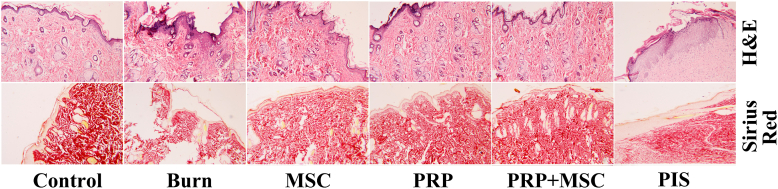
Effects of bone marrow derived-mesenchymal stem cells (BMSCs), platelet-rich plasma (PRP), BMSCs + PRP, or standard treatment (PIS) on the histological features by **(A)** H & E stain and **(B)** Sirius red in a full-thickness skin burns in rats. (100x).

In a histological view, the staining of skin tissue from the control group with H&E showed normal skin dermis and epidermis. However, the burned tissues revealed diffuse inflammatory cell infiltration, and a small number of blood vessels without any signs of epithelization. On the other hand, BMSCs, PRP, or both displayed marked epithelization, granulation tissue formation, less inflammatory cells, and more blood capillaries than the untreated burn group. Further, via Sirius red staining, normal collagen bundles were observed in the dermis. In contrast, minimal collagen deposition in untreated burns while the different therapies resulted in denser, more arranged, and mature collagen (Fig. 7).

## 4 Discussion

Based on previous studies [24,25], BMSCs and PRP therapy successfully achieved promising outcomes in the regenerative process during the healing of cutaneous wounds. In this study, we used PRP along with BMSCs in order to maximize the effect of these cells in a rat model of a full-thickness skin burn injury. A substantial decrease in the burn area and epithelization time with an increase in burn contracture rate was observed in the burn group received by both agents. The results obtained by Ghufran et al. [26] demonstrated that rats treated with curcumin-preconditioned BMSCs and/or PRP showed earlier wound closure as compared to the other groups. Similarly, microvesicles derived from BMSCs and PRP significantly decreased burn wound size after two weeks from burn induction compared to the burn group that received only PIS (traditional treatment) [27]. Further, re-epithelialization is a key process for competent wound closure. Previously, autologous PRP was found to accelerate re-epithelialization by increasing the proliferation and differentiation of epidermal cells on days 3, 5, and 7 post-burn [28]. BMSCs administration to rats with induced oral ulcers elicited better histological consequences and high rate of epithelial cell migration with complete re-epithelization on day 10 [29]. Hence, it could be presumed that BMSCs and/or PRP accomplished, in combination, better healing outcomes in a shorter time.

Angiogenesis is the process of forming new blood vessels that is extremely important for wound repair after injury. In addition, it is a prerequisite for the formation of granulation tissue to replenish the damaged area [30]. VEGF is one of the most potent angiogenic mediators that are involved in the regulation of vascular permeability, and enhancement of cellular migration, proliferation and survival [31].

Previous studies have addressed the ability of BMSCs to promote vascular angiogenesis in damaged tissues. For instance, preconditioned MSCs with quercetin and rutin resulted in notable upregulation in the gene expression of VEGF and increased the number of blood vessels in cold-induced burn wounds [25]. Also, BMSCs-derived exosome administration evoked noteworthy elevations in VEGF, epidermal growth factor (EGF), and their receptors (VEGF-R2, VEGF-R3) in rats with subcutaneous fat grafting [31]. Our findings are also in agreement with the previous study that reported the impact of PRP injection elevated the levels of VEGF in a rat model of deep dermal burn injury [32]. Topical application of PRP gel and adipose tissue-derived SCs improved the skin graft texture by increasing the expression of VEGF, fibroblast growth factor (BFGF), and platelet-derived growth factor subunit B (PDGFB) with consequent improvement in skin neovascularization [33]. These findings proved the pro-angiogenic effects of our combined therapy by stimulating endothelial cell functions that are required for the formation of new blood vessels.

Interestingly, combined cell therapy with PRP considerably improved the scar regulating parameters such as MMP-9 and TGF-β1 levels as well as the immune reaction of TGF-β1 in the burn area. TGF-β1, a growth factor, promotes the growth of tissue fibrosis that also regulates the activity of MMPs such as MMP9 and MMP2 [34]. These proteolytic enzymes are mainly distributed in scar tissue and are responsible for the remodeling of extracellular matrix and collagen [27]. Former studies have suggested that modulation of the TGF-β signaling pathway was able to block tissue scarring in various wound models [35,36]. Our results are in harmony with former authors who reported that subcutaneous injection of the burned rats with BM-MSCs (2 × 10^6^ cells/ml) elicited notable downregulation in TGF-β and MMP-9 [37]. The combined therapy with microvesicles from BMSCs and PRP evoked notable downregulation in the protein expression of MMP-3 and TGF-β in a murine burn model [27]. Furthermore, Cui and colleagues found that PRP provided significant repairing effects on photoaged HaCaT keratinocytes either being applied before or after UVB-irradiation via downregulation of MMP1 and MMP9 expression levels [38]. From the previous findings, it could be concluded that the co-administration of BMSCs and PRP is helpful to achieve scarless burn wound healing.

Importantly, other factors like oxidizing environment may seriously disrupt the process of wound vascularization, fibrosis, and remodeling, and therapy hinders the success of the healing process [39]. An earlier study has shown that levels of MDA were elevated and the antioxidant activities of SOD, CAT, and GPx were decreased in the wound area of diabetic rats [40]. In agreement with former authors [27,41], both tested agents were capable to combat the burn injury-induced oxidative stress in the rat model. Oztan et al. [42] identified the potential of topically applied PRP against the oxidant status associated with esophageal stricture after induction of experimental corrosive burn.

Importantly, other factors like oxidizing environment may seriously disrupt the process of wound vascularization, fibrosis and remodeling, therapy hinder the success of the healing process [39]. Earlier study has shown that levels of MDA were elevated and the antioxidant activities of SOD, CAT and GPx were decreased in the wound area of diabetic rats [40]. In agreement with former authors [27,41], both tested agents were capable to combat the burn injury-induced oxidative stress in the rat model. Oztan et al. [42] identified the potential of topically applied PRP against the oxidant status associated with esophageal stricture after induction of experimental corrosive burn. Similar results were also obtained after *in vitro* application of PRP therapy in photoaged HaCaT cells [38]. Moreover, Chen et al. [43] verified the antioxidant effect of PRP in the promotion of diabetic ulcer repair by elevating the SOD content and decreasing the MDA contents. In parallel, BMSCs application decreased the amount of MDA, and increased SOD activity with consequent enhancement of the flap survival rate [41]. The antioxidant power of BMSCs may endorse their activation of Nrf2/HO-1 signaling pathway in the wound area that was supported by previous reports [27,39,44].

The balance between the pro-inflammatory and the anti-inflammatory mediators at the site of wounds has an irreplaceable role in the wound-repairing process [26]. Our data demonstrated dramatic decreases in the tissue levels of IL-6, and TNF-α as well as the levels and immune reaction of Nf-κB following the treatment with BMSCs and PRP. In agreement with the current results, Irfan et al. [25] have reported that hUC-MSCs preconditioned with quercetin and rutin attenuated the wound inflammation by decreasing the mRNA expression of pro-inflammatory cytokines (IL-1β and IL-6) besides upregulation of anti-inflammatory members (IL-5 and IL-4). In the same regard, BMSCs-derived exosomes were found to downregulate TNF-α and IL-1β in the wound area in diabetic rats [44]. A study reported by Zheng et al. [30] showed that MSCs could reduce the levels of TNF-α and IFN-γ, decrease the number of inflammatory cells, and upregulate the gene expression of IL-10 in cutaneous wounds. The immunomodulating and anti-inflammatory properties of BMSCs were also demonstrated by former studies that indicated their potential contribution for successful wound healing [27,37,41,45]. In addition, the anti-inflammatory efficacy of PRP was clarified by former reports on experimental burn models [27,28,46]. Pretreatment with PRP exerted a protective effect against inflammation provoked by UVB in HaCaT cells [38]. Further, Chen et al. [43] attenuated the inflammatory cytokines; IL-1β, IL-10, and NLRP3 in the granulation tissue of diabetic ulcerated wound in rats.

In addition to these findings, we observed significant declines in burn NO levels and iNOS gene expression in the burn groups that received BMSCs and PRP. Upon being triggered by tissue cytokines, iNOS is stimulated to produce excess NO which plays important role in the inflammatory process [47,48]. Gong and colleagues [49] have reported that induced severe burn injury induced the expression of iNOS in the serum, and intestine of injured rats. Additionally, iNOS contributed to cardiac oxidative stress and inflammation caused by burn injury in a mouse model [50,51]. Topically applied PRP subsided the wound inflammation by decreasing the levels of tissue cytokines and iNOS expression in infected skin wounds in rats [52]. The exosomes from ADSCs decreased the protein expression of iNOS in both *in vitro* (mouse RAW264.7 cells) and *in vivo* (skin wound in mice) investigations [53]. These results indicated the co-administered agents achieved recovery from tissue inflammation that leads to fastened wound healing with reduced scarring.

Earlier studies on animal models found that after burn induction, the expression of apoptotic biomarkers gradually increased in the skin cells [54,55]. Our results showed that BMSCs administration significantly counteracted burn injury-induced apoptosis in skin cells. The treatment with hUCMSCs provoked notable anti-apoptotic effects in the heart, kidney, and liver caused by severe burns in rats [56]. The Cas-3-positive stained cells showed marked decreases in the corneal tissue of experimental rats that received BMSCs derived from bone marrow and adipose tissues in an alkaline burn model [57]. hAMSCs and their conditioned medium displayed noteworthy *in vitro* anti-apoptotic action in HaCAT and DFL cells exposed to heat stress by activating PI3K/AKT signaling pathway [58]. In addition, PRP treatment repressed apoptotic cell death in irradiated HaCaT cells by increasing the Bcl-2 expression levels and decreasing the Bax levels [38]. Tsai et al. [59] evaluated the cell apoptosis of injured gastrocnemius muscles by TUNEL assay that was treated with PRP released and they found marked decreases in the apoptotic cells in injured muscles of rats treated with PRP. Likewise, Hydrogels loaded with PRP suppressed the fibroblast apoptosis that was mediated via the activation of the adenosine A2A receptor in rats [60]. All of the abovementioned results indicated that PRP and BMSCs reversed burn injury-induced apoptosis in skin cells with consequent accelerated wound closure.

## 5 Conclusions

Overall, the current study substantiated that BMSCs and/or PRP administration evoked notable improvement in burn healing by increasing the contraction rate, burn area, and period of epithelization. Besides, both therapeutic agents markedly countered the oxidative damage and the inflammatory reaction in the burn-injured skin in addition to the apoptotic mediators. It could be presumed that BMSCs + PRP worked proficiently to up-regulate the angiogenic process via the regeneration of VEGF. Noteworthy improvement was noticed in tissue remodeling was achieved by the BMSCs and PRP by increasing collagen deposition and elevating MMP9 and TGF-β levels. Thus, it could be envisioned that combined cell-based therapy with PRP is promising to boost the healing of cutaneous burn injuries.

## Author contribution statement

1 - Conceived and designed the experiments;

2 - Performed the experiments;

3 - Analyzed and interpreted the data;

4 - Contributed reagents, materials, analysis tools or data;

5 - Wrote the paper.

## Funding statement

Princess Nourah bint Abdulrahman University Researchers Supporting Project number (PNURSP2023R23), 10.13039/501100004242Princess Nourah bint Abdulrahman University, Riyadh, Saudi Arabia.

## Institutional review board statement

The European Community Directive (86/609/EEC) was followed in conducting the current experimental method. The Institutional Animal Ethics Committee accepted the animal care procedures at the Zoology and Entomology Department, Faculty of Science, Helwan University, and they complied with the National Institutes of Health (NIH) Guidelines for the Care and Use of Laboratory Animals, 8th edition. (Approval Number: HU2021/Z/AEB1221-01).

## Informed Consent Statement

Not applicable.

## Data availability statement

All the relevant data are within the paper.

## Declaration of competing interest

The authors declare that they have no known competing financial interests or personal relationships that could have appeared to influence the work reported in this paper.
